# Supplementary material for: Comparative efficacy of opioid and non-opioid analgesics in labor pain management: A network meta-analysis
Source: PLoS One. 2024 Jun 18;19(6):e0303174. doi: 10.1371/journal.pone.0303174 (PMC11185472; doi:10.1371/journal.pone.0303174)
Supplement: S1 File — (PDF) [file pone.0303174.s001.pdf]

S1. Search policy:

The keywords related to Population and Intervention were used for a PubMed search, resulting in the retrieval of 97 records.

1. Population

(((((Pain, Labor[Title/Abstract]) OR (Obstetric Pain[Title/Abstract])) OR (Pain, Obstetric[Title/Abstract])) OR ("Labor Pain"[Mesh])) OR (("Analgesia, Obstetrical"[Mesh]) OR (((Obstetrical Analgesia[Title/Abstract]) OR (Analgesia, Obstetric[Title/Abstract])) OR (Obstetric Analgesia[Title/Abstract])))) OR (("Analgesia, Epidural"[Mesh]) OR (Epidural Analgesia[Title/Abstract]))

2. Intervention

(((((((3-(4-Methoxycarbonyl-4-((1-oxopropyl)phenylamino)-1-piperidine)propanoic Acid Methyl Ester[Title/Abstract]) OR (Remifentanil Hydrochloride[Title/Abstract])) OR (Ultiva[Title/Abstract])) OR (Remifentanil Monohydrochloride[Title/Abstract])) OR (GI 87084B[Title/Abstract])) OR (GI 87084B[Title/Abstract])) OR (GI-87084B[Title/Abstract])) OR ("Remifentanil"[Mesh])

Using similar search strategies, 212, 98 and 226 records were obtained from EMBASE, Cochrane and WOS.
