# Supplementary material for: Comparative efficacy of opioid and non-opioid analgesics in labor pain management: A network meta-analysis
Source: PLoS One. 2024 Jun 18;19(6):e0303174. doi: 10.1371/journal.pone.0303174 (PMC11185472; doi:10.1371/journal.pone.0303174)
Supplement: S2 File — (PDF) [file pone.0303174.s002.pdf]

S2. Additional Tables and Figures

Study ID	D1	D2	D3	D4	D5	Overall
Tveit 2012	+	!	+	+	+	!
Evron 2005	+	+	+	+	+	+
Douma 2010	+	+	+	+	+	+
Stocki 2014	!	+	+	+	+	!
Douma 2011	+	+	!	!	+	!
Ismail 2012	+	+	+	+	+	+
Jia 2020	!	!	+	!	+	!
Rezk 2015	+	+	+	+	+	+
Nunes 2019	+	+	+	+	+	+
Zhao 2017	+	+	+	+	+	+
Wang 2018	!	!	+	!	+	!
Karadjova 2019	+	!	+	!	+	!
Li 2020	+	+	+	+	+	+
Cheng 2019	+	+	+	+	+	+
Wu 2022	!	!	+	!	+	!

\+ Low risk

! Some concerns

\- High risk

D1 Randomisation process

D2 Deviations from the intended interventions

D3 Missing outcome data

D4 Measurement of the outcome

D5 Selection of the reported result

S2 Fig 1. Analysis of the included clinical trials on labor analgesia using the ROB2 risk bias assessment tool. Low risk, some concerns and high risk were evaluated from five aspects: D1,D2,D3,D4 and D5, and the overall risk assessment was finally summarized.

Compared with placebo	OR	95%Crl
**Drug category**		
Combination	5.79	(3.80, 7.82)
Nonopioid	5.6	(2.88, 8.24)
Opioid	4.45	(2.21, 6.59)
**Individual treatment**		
Bupivacain + Fentanyl	5.87	(1.80, 9.96)
Dexmedetomidine + Ropivacaine	6.44	(2.50, 10.41)
Dexmedetomidine + Ropivacaine + Sufentanil	7.29	(2.49, 11.99)
Dipyrone	4.53	(-1.38, 10.48)
Fentanyl	4.39	(-0.23, 9.16)
Fentanyl + Ropivacaine	6.37	(0.90, 11.77)
Pethidine	4.44	(0.18, 8.81)
Remifentanil	4.39	(1.03, 7.72)
Ropivacaine	4.99	(0.09, 9.88)
Ropivacaine + Sufentanil	5.84	(3.13, 8.62)

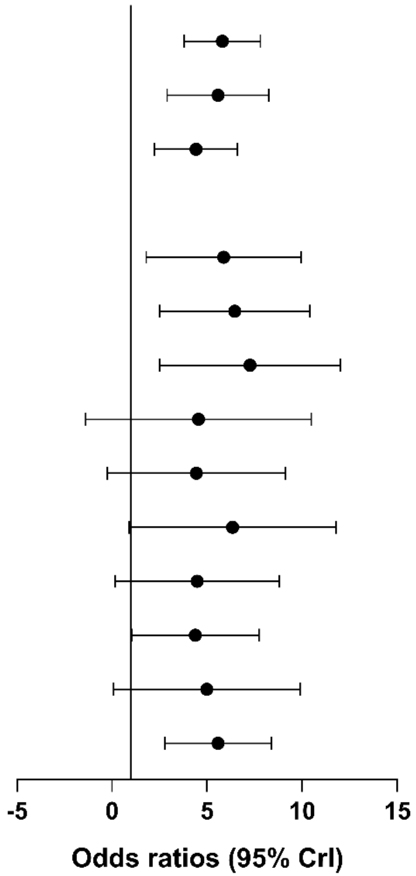

S2 Fig 2. Forest plot of odds ratios of pain scores. Multiple drugs were compared with placebo.

Compared with placebo	OR	95%CrI
**Drug category**		
Combination	2.34	(0.08, 170.13)
Nonopioid	0.85	(0.02, 73.89)
Opioid	2.62	(0.18, 123.69)
**Individual treatment**		
Bupivacain + Fentanyl	1.9	(0.12, 50.33)
Dexmedetomidine + Ropivacaine	0.09	(0.00, 4.78)
Dexmedetomidine + Ropivacaine + Sufentanil	0.19	(0.00, 20.36)
Dipyrone	7.02	(0.07, 862.86)
Fentanyl	2.98	(0.16, 98.80)
Fentanyl + Ropivacaine	2.36	(0.11, 111.85)
Pethidine	5.69	(0.37, 205.12)
Remifentanil	3.27	(0.24, 82.00)
Ropivacaine	0.18	(0.00, 11.63)
Ropivacaine + Sufentanil	0.76	(0.06, 26.18)

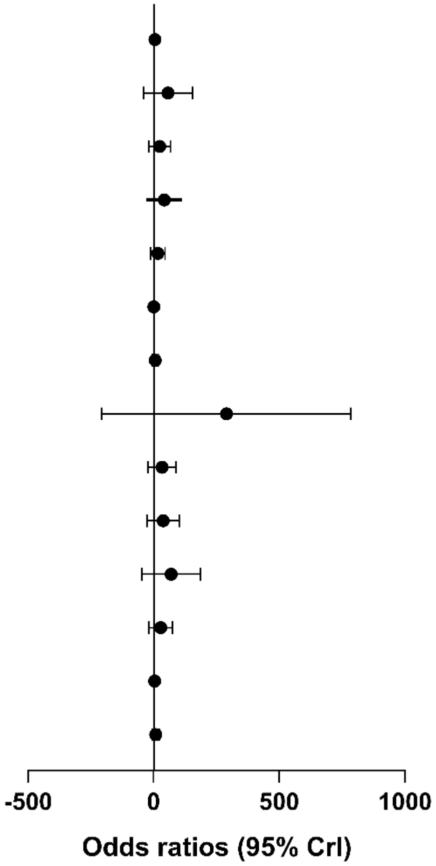

S2 Fig 3. Forest plot of odds ratios of side effects(Nausea and Vomiting). Multiple drugs were compared with placebo.

S2 Table 1. Network meta-analysis consistency model of pain scores classified by drug category.

Combination	-0.20 (-2.08, 1.55)	-1.34 (-2.54, -0.18)	-5.79 (-7.82, -3.80)
0.20 (-1.55, 2.08)	Nonopioid	-1.13 (-3.07, 0.78)	-5.60 (-8.24, -2.88)
1.34 (0.18, 2.54)	1.13 (-0.78, 3.07)	Opioid	-4.45 (-6.59, -2.21)
5.79 (3.80, 7.82)	5.60 (2.88, 8.24)	4.45 (2.21, 6.59)	Placebo

S2 Table 2. Network meta-analysis inconsistency model of pain scores classified by drug category.

Combination	0.33 (-1.19, 1.72)	-1.59 (-2.54, -0.72)	-4.33 (-6.46, -2.34)
-0.33 (-1.72, 1.19)	Nonopioid	-0.20 (-2.19, 1.59)	-5.67 (-8.14, -3.26)
1.59 (0.72, 2.54)	0.20 (-1.59, 2.19)	Opioid	-5.47 (-7.19, -3.55)
4.33 (2.34, 6.46)	5.67 (3.26, 8.14)	5.47 (3.55, 7.19)	Placebo

S2 Table 3. Network meta-analysis consistency model of pain scores classified by individual treatment.

Bupivacain + Fentanyl	0.58 (-4.02, 5.18)	1.40 (-3.81, 6.60)	-1.35 (-6.72, 4.11)	-1.48 (-5.49, 2.67)	0.49 (-4.41, 5.29)	-1.44 (-5.01, 2.16)	-1.51 (-3.85, 0.79)	-0.90 (-6.33, 4.52)	-0.03 (-3.67, 3.58)	-5.87 (-9.96, -1.80)
-0.58 (-5.18, 4.02)	Dexmedetomidine + Ropivacaine	0.85 (-2.98, 4.59)	-1.94 (-8.21, 4.37)	-2.08 (-7.13, 3.11)	-0.09 (-6.04, 5.72)	-2.02 (-6.80, 2.87)	-2.09 (-6.04, 1.92)	-1.48 (-4.30, 1.40)	-0.62 (-3.43, 2.27)	-6.44 (-10.41, -2.50)
-1.40 (-6.60, 3.81)	-0.85 (-4.59, 2.98)	Dexmedetomidine + Ropivacaine + Sufentanil	-2.78 (-9.46, 4.05)	-2.88 (-8.53, 2.88)	-0.92 (-7.32, 5.61)	-2.85 (-8.18, 2.60)	-2.93 (-7.55, 1.77)	-2.32 (-7.06, 2.42)	-1.47 (-5.18, 2.37)	-7.29 (-11.99, -2.49)
1.35 (-4.11, 6.72)	1.94 (-4.37, 8.21)	2.78 (-4.05, 9.46)	Dipyrone	-0.11 (-5.09, 4.77)	1.87 (-4.74, 8.40)	-0.06 (-4.15, 3.86)	-0.15 (-5.08, 4.71)	0.46 (-6.49, 7.32)	1.31 (-4.40, 6.96)	-4.53 (-10.48, 1.38)
1.48 (-2.67, 5.49)	2.08 (-3.11, 7.13)	2.88 (-2.88, 8.53)	0.11 (-4.77, 5.09)	Fentanyl	1.98 (-3.58, 7.41)	0.06 (-2.74, 2.79)	-0.03 (-3.40, 3.31)	0.61 (-5.29, 6.37)	1.44 (-2.94, 5.84)	-4.39 (-9.16, 0.23)
-0.49 (-5.29, 4.41)	0.09 (-5.72, 6.04)	0.92 (-5.61, 7.32)	-1.87 (-8.40, 4.74)	-1.98 (-7.41, 3.58)	Fentanyl + Ropivacaine	-1.94 (-7.01, 3.18)	-2.00 (-6.26, 2.30)	-1.40 (-7.82, 5.13)	-0.54 (-5.66, 4.68)	-6.37 (-11.77, -0.90)
1.44 (-2.16, 5.01)	2.02 (-2.87, 6.80)	2.85 (-2.60, 8.18)	0.06 (-3.86, 4.15)	-0.06 (-2.79, 2.74)	1.94 (-3.18, 7.01)	Pethidine	-0.07 (-2.89, 2.71)	0.55 (-5.10, 6.03)	1.40 (-2.57, 5.35)	-4.44 (-8.81, -0.18)
1.51 (-0.79, 3.85)	2.09 (-1.92, 6.04)	2.93 (-1.77, 7.55)	0.15 (-4.71, 5.08)	0.03 (-3.31, 3.40)	2.00 (-2.30, 6.26)	0.07 (-2.71, 2.89)	Remifentanil	0.63 (-4.29, 5.44)	1.46 (-1.33, 4.28)	-4.39 (-7.72, -1.03)
0.90 (-4.52, 6.33)	1.48 (-1.40, 4.30)	2.32 (-2.42, 7.06)	-0.46 (-7.32, 6.49)	-0.61 (-6.37, 5.29)	1.40 (-5.13, 7.82)	-0.55 (-6.03, 5.10)	-0.63 (-5.44, 4.29)	Ropivacaine	0.84 (-3.10, 4.93)	-4.99 (-9.88, -0.09)
0.03 (-3.58, 3.67)	0.62 (-2.27, 3.43)	1.47 (-2.37, 5.18)	-1.31 (-6.96, 4.40)	-1.44 (-5.84, 2.94)	0.54 (-4.68, 5.66)	-1.40 (-5.35, 2.57)	-1.46 (-4.28, 1.33)	-0.84 (-4.93, 3.10)	Ropivacaine + Sufentanil	-5.84 (-8.62, -3.13)
5.87 (1.80, 9.96)	6.44 (2.50, 10.41)	7.29 (2.49, 11.99)	4.53 (-1.38, 10.48)	4.39 (-0.23, 9.16)	6.37 (0.90, 11.77)	4.44 (0.18, 8.81)	4.39 (1.03, 7.72)	4.99 (0.09, 9.88)	5.84 (3.13, 8.62)	Placebo

S2 Table 4. Network meta-analysis inconsistency model of pain scores classified by individual treatment.

Bupivacain + Fentanyl	0.99 (-2.24, 3.88)	1.80 (-1.66, 5.23)	0.41 (-4.03, 3.87)	-0.28 (-3.38, 2.38)	0.56 (-2.79, 3.84)	0.33 (-2.97, 2.92)	-1.47 (-2.96, -0.01)	-0.51 (-4.17, 2.88)	0.31 (-2.14, 2.69)	-4.05 (-7.81, -1.07)
-0.99 (-3.88, 2.24)	Dexmedetomidine + Ropivacaine	0.82 (-1.56, 3.36)	-0.58 (-5.37, 3.57)	-1.25 (-4.95, 2.19)	-0.42 (-4.38, 3.78)	-0.63 (-4.53, 2.66)	-2.46 (-5.01, 0.37)	-1.50 (-3.33, 0.35)	-0.65 (-2.42, 1.27)	-5.02 (-8.32, -2.18)
-1.80 (-5.23, 1.66)	-0.82 (-3.36, 1.56)	Dexmedetomidine + Ropivacaine + Sufentanil	-1.41 (-6.60, 2.92)	-2.09 (-6.15, 1.57)	-1.25 (-5.54, 2.97)	-1.47 (-5.76, 2.12)	-3.28 (-6.37, -0.14)	-2.33 (-5.45, 0.61)	-1.49 (-3.97, 0.99)	-5.85 (-9.65, -2.69)
-0.41 (-3.87, 4.03)	0.58 (-3.57, 5.37)	1.41 (-2.92, 6.60)	Dipyrone	-0.71 (-3.62, 2.73)	0.13 (-4.14, 5.33)	-0.06 (-2.63, 2.58)	-1.89 (-5.09, 2.30)	-0.92 (-5.32, 4.16)	-0.07 (-3.73, 4.32)	-4.46 (-8.87, 0.24)
0.28 (-2.38, 3.38)	1.25 (-2.19, 4.95)	2.09 (-1.57, 6.15)	0.71 (-2.73, 3.62)	Fentanyl	0.83 (-2.86, 5.00)	0.64 (-1.44, 2.34)	-1.19 (-3.51, 1.52)	-0.24 (-4.15, 3.85)	0.60 (-2.22, 3.79)	-3.78 (-7.51, -0.12)
-0.56 (-3.84, 2.79)	0.42 (-3.78, 4.38)	1.25 (-2.97, 5.54)	-0.13 (-5.33, 4.14)	-0.83 (-5.00, 2.86)	Fentanyl + Ropivacaine	-0.20 (-4.58, 3.31)	-2.03 (-5.02, 0.93)	-1.09 (-5.71, 3.33)	-0.24 (-3.86, 3.33)	-4.61 (-9.24, -0.59)
-0.33 (-2.92, 2.97)	0.63 (-2.66, 4.53)	1.47 (-2.12, 5.76)	0.06 (-2.58, 2.63)	-0.64 (-2.34, 1.44)	0.20 (-3.31, 4.58)	Pethidine	1.70 (-1.21, 3.98)	-0.87 (-4.59, 3.41)	-0.01 (-2.80, 3.37)	-4.40 (-8.05, -0.63)
1.47 (0.01, 2.96)	2.46 (-0.37, 5.01)	3.28 (0.14, 6.37)	1.89 (-2.30, 5.09)	1.19 (-1.52, 3.51)	2.03 (-0.93, 5.02)	-1.70 (-3.98, 1.21)	Remifentanil	0.97 (-2.36, 3.96)	1.79 (-0.12, 3.63)	-5.32 (-7.55, -2.71)
0.51 (-2.88, 4.17)	1.50 (-0.35, 3.33)	2.33 (-0.61, 5.45)	0.92 (-4.16, 5.32)	0.24 (-3.85, 4.15)	1.09 (-3.33, 5.71)	0.87 (-3.41, 4.59)	-0.97 (-3.96, 2.36)	Ropivacaine	0.84 (-1.63, 3.48)	-3.52 (-7.32, -0.16)
-0.31 (-2.69, 2.14)	0.65 (-1.27, 2.42)	1.49 (-0.99, 3.97)	0.07 (-4.32, 3.73)	-0.60 (-3.79, 2.22)	0.24 (-3.33, 3.86)	0.01 (-3.37, 2.80)	-1.79 (-3.63, 0.12)	-0.84 (-3.48, 1.63)	Ropivacaine + Sufentanil	-4.36 (-7.05, -2.17)
4.05 (1.07, 7.81)	5.02 (2.18, 8.32)	5.85 (2.69, 9.65)	4.46 (-0.24, 8.87)	3.78 (0.12, 7.51)	4.61 (0.59, 9.24)	4.40 (0.63, 8.05)	5.32 (2.71, 7.55)	3.52 (0.16, 7.32)	4.36 (2.17, 7.05)	Placebo

S2 Table 5. Network meta-analysis consistency model of side effects(Nausea and Vomiting) classified by drug category.

Combination	0.20 (0.04, 1.08)	1.30 (0.06, 10.38)	0.43 (0.01, 12.62)
4.89 (0.92, 28.12)	Nonopioid	3.63 (0.21, 39.98)	1.18 (0.01, 55.55)
0.77 (0.10, 17.19)	0.28 (0.03, 4.76)	Opioid	0.38 (0.01, 5.51)
2.34 (0.08, 170.13)	0.85 (0.02, 73.89)	2.62 (0.18, 123.69)	Placebo

S2 Table 6. Network meta-analysis inconsistency model of side effects(Nausea and Vomiting) classified by drug category.

Combination	0.19 (0.03, 1.03)	1.22 (0.10, 7.29)	0.38 (0.01, 10.96)
5.39 (0.97, 30.58)	Nonopioid	4.12 (0.28, 90.20)	1.26 (0.02, 155.41)
0.82 (0.14, 9.75)	0.24 (0.01, 3.53)	Opioid	0.41 (0.00, 5.28)
2.61 (0.09, 115.58)	0.79 (0.01, 47.78)	2.42 (0.19, 209.28)	Placebo

S2 Table 7. Network meta-analysis consistency model of side effects(Nausea and Vomiting) classified by individual treatment.

Bupivacain + Fentanyl	0.05 (0.00, 0.83)	0.10 (0.00, 4.42)	4.03 (0.06, 149.19)	1.52 (0.38, 8.91)	1.29 (0.17, 12.74)	3.06 (0.86, 18.91)	1.65 (0.78, 4.57)	0.10 (0.00, 2.21)	0.45 (0.06, 3.75)	0.53 (0.02, 8.10)
20.39 (1.20, 418.16)	Dexmedetomidine + Ropivacaine	2.03 (0.04, 54.26)	72.16 (0.59, 7251.86)	31.57 (1.72, 787.37)	25.25 (1.12, 1092.13)	60.43 (3.81, 1669.71)	34.07 (2.62, 647.45)	1.87 (0.49, 7.26)	8.22 (1.50, 78.07)	11.67 (0.21, 324.19)
10.49 (0.23, 592.75)	0.49 (0.02, 27.57)	Dexmedetomidine + Ropivacaine + Sufentanil	40.55 (0.13, 5923.20)	14.86 (0.35, 1199.98)	13.76 (0.21, 1187.07)	31.69 (0.77, 2296.52)	16.43 (0.48, 982.96)	0.89 (0.03, 65.75)	4.14 (0.30, 173.90)	5.37 (0.05, 597.73)
0.25 (0.01, 15.49)	0.01 (0.00, 1.69)	0.02 (0.00, 7.52)	Dipyrone	0.39 (0.01, 22.98)	0.37 (0.01, 30.79)	0.82 (0.03, 43.98)	0.42 (0.01, 25.51)	0.02 (0.00, 4.06)	0.13 (0.00, 11.40)	0.14 (0.00, 15.16)
0.66 (0.11, 2.67)	0.03 (0.00, 0.58)	0.07 (0.00, 2.83)	2.57 (0.04, 81.08)	Fentanyl	0.83 (0.08, 8.55)	1.89 (0.76, 6.53)	1.07 (0.28, 3.91)	0.06 (0.00, 1.50)	0.28 (0.03, 2.56)	0.34 (0.01, 6.16)
0.78 (0.08, 5.96)	0.04 (0.00, 0.89)	0.07 (0.00, 4.69)	2.67 (0.03, 143.52)	1.21 (0.12, 12.85)	Fentanyl + Ropivacaine	2.39 (0.25, 26.39)	1.33 (0.17, 8.82)	0.08 (0.00, 2.22)	0.31 (0.02, 4.85)	0.42 (0.01, 8.82)
0.33 (0.05, 1.16)	0.02 (0.00, 0.26)	0.03 (0.00, 1.31)	1.23 (0.02, 32.15)	0.53 (0.15, 1.31)	0.42 (0.04, 3.95)	Pethidine	0.26 (0.04, 1.33)	0.03 (0.00, 0.68)	0.14 (0.01, 1.35)	0.18 (0.00, 2.67)
0.61 (0.22, 1.29)	0.03 (0.00, 0.38)	0.06 (0.00, 2.10)	2.36 (0.04, 75.25)	0.93 (0.26, 3.59)	0.75 (0.11, 5.74)	3.81 (0.75, 28.02)	Remifentanil	0.06 (0.00, 1.06)	0.27 (0.04, 1.73)	0.31 (0.01, 4.10)
10.04 (0.45, 311.19)	0.54 (0.14, 2.04)	1.12 (0.02, 36.02)	40.10 (0.25, 4375.09)	17.72 (0.67, 556.97)	12.90 (0.45, 851.95)	31.69 (1.47, 1164.20)	16.41 (0.94, 512.07)	Ropivacaine	4.42 (0.50, 57.80)	5.51 (0.09, 252.34)
2.24 (0.27, 15.80)	0.12 (0.01, 0.67)	0.24 (0.01, 3.30)	7.70 (0.09, 479.56)	3.51 (0.39, 33.58)	3.19 (0.21, 44.93)	7.17 (0.74, 67.28)	3.70 (0.58, 23.11)	0.23 (0.02, 2.00)	Ropivacaine + Sufentanil	1.32 (0.04, 17.37)
1.90 (0.12, 50.33)	0.09 (0.00, 4.78)	0.19 (0.00, 20.36)	7.02 (0.07, 862.86)	2.98 (0.16, 98.80)	2.36 (0.11, 111.85)	5.69 (0.37, 205.12)	3.27 (0.24, 82.00)	0.18 (0.00, 11.63)	0.76 (0.06, 26.18)	Placebo

S2 Table 8. Network meta-analysis inconsistency model of side effects(Nausea and Vomiting) classified by individual treatment.

Bupivacain + Fentanyl	0.05 (0.00, 0.88)	0.09 (0.00, 3.33)	3.16 (0.07, 227.10)	1.46 (0.37, 9.89)	1.42 (0.16, 13.84)	2.93 (0.85, 20.63)	1.59 (0.75, 4.84)	0.09 (0.01, 2.09)	0.45 (0.05, 3.71)	0.51 (0.01, 10.26)
19.68 (1.14, 308.97)	Dexmedetomidine + Ropivacaine	2.37 (0.07, 40.00)	80.72 (0.66, 8392.61)	32.66 (1.82, 558.98)	26.07 (1.06, 718.38)	62.19 (4.02, 1273.72)	32.73 (2.63, 451.66)	1.77 (0.49, 7.04)	9.19 (1.52, 50.74)	12.13 (0.14, 307.98)
10.63 (0.30, 382.32)	0.42 (0.02, 14.73)	Dexmedetomidine + Ropivacaine + Sufentanil	27.33 (0.20, 9512.20)	14.97 (0.48, 747.56)	15.75 (0.28, 889.42)	27.13 (1.04, 1671.12)	17.05 (0.61, 623.34)	0.74 (0.04, 32.74)	4.76 (0.28, 99.65)	5.89 (0.04, 410.77)
0.32 (0.00, 14.20)	0.01 (0.00, 1.52)	0.04 (0.00, 4.89)	Dipyrone	0.55 (0.01, 19.49)	0.40 (0.00, 28.50)	1.01 (0.02, 37.04)	0.50 (0.01, 21.48)	0.02 (0.00, 3.26)	0.15 (0.00, 9.68)	0.22 (0.00, 15.57)
0.68 (0.10, 2.73)	0.03 (0.00, 0.55)	0.07 (0.00, 2.08)	1.82 (0.05, 116.09)	Fentanyl	0.88 (0.07, 9.09)	1.92 (0.72, 6.46)	1.10 (0.27, 3.83)	0.05 (0.00, 1.37)	0.32 (0.02, 2.72)	0.39 (0.01, 7.58)
0.70 (0.07, 6.16)	0.04 (0.00, 0.94)	0.06 (0.00, 3.53)	2.49 (0.04, 264.43)	1.13 (0.11, 13.71)	Fentanyl + Ropivacaine	2.30 (0.24, 31.60)	1.22 (0.16, 9.14)	0.07 (0.00, 2.09)	0.35 (0.02, 5.35)	0.35 (0.01, 11.48)
0.34 (0.05, 1.17)	0.02 (0.00, 0.25)	0.04 (0.00, 0.96)	0.99 (0.03, 45.86)	0.52 (0.15, 1.40)	0.44 (0.03, 4.12)	Pethidine	0.32 (0.04, 1.32)	0.03 (0.00, 0.62)	0.17 (0.01, 1.12)	0.20 (0.00, 4.10)
0.63 (0.21, 1.33)	0.03 (0.00, 0.38)	0.06 (0.00, 1.65)	1.99 (0.05, 123.10)	0.91 (0.26, 3.69)	0.82 (0.11, 6.42)	3.08 (0.76, 25.47)	Remifentanil	0.06 (0.00, 0.94)	0.29 (0.04, 1.57)	0.36 (0.01, 4.90)
10.75 (0.48, 187.24)	0.57 (0.14, 2.05)	1.36 (0.03, 26.43)	40.89 (0.31, 5534.16)	18.35 (0.73, 368.94)	14.39 (0.48, 462.63)	34.79 (1.61, 809.59)	17.53 (1.06, 273.45)	Ropivacaine	4.95 (0.53, 41.26)	6.87 (0.07, 228.51)
2.20 (0.27, 19.25)	0.11 (0.02, 0.66)	0.21 (0.01, 3.54)	6.61 (0.10, 769.63)	3.15 (0.37, 42.58)	2.87 (0.19, 49.58)	5.89 (0.89, 83.54)	3.50 (0.64, 27.59)	0.20 (0.02, 1.90)	Ropivacaine + Sufentanil	1.31 (0.02, 27.54)
1.97 (0.10, 74.38)	0.08 (0.00, 7.13)	0.17 (0.00, 25.71)	4.56 (0.06, 1730.86)	2.56 (0.13, 154.47)	2.82 (0.09, 157.71)	5.01 (0.24, 310.38)	2.82 (0.20, 114.60)	0.15 (0.00, 15.05)	0.77 (0.04, 41.37)	Placebo
