# Supplementary material for: Comparative efficacy of opioid and non-opioid analgesics in labor pain management: A network meta-analysis
Source: PLoS One. 2024 Jun 18;19(6):e0303174. doi: 10.1371/journal.pone.0303174 (PMC11185472; doi:10.1371/journal.pone.0303174)

PRISMA NMA Checklist of Items to Include When Reporting A Systematic Review Involving a Network Meta-analysis

Section/Topic	Item #	Checklist Item	Reported on Page #
**TITLE**		**Comparative Efficacy of Opioid and Non-Opioid Analgesics in Labor Pain Management: A Network Meta-Analysis**	
Title	1	Identify the report as a systematic review *incorporating a network meta-analysis (or related form of meta-analysis).*	1
**ABSTRACT**			
Structured summary	2	Provide a structured summary including, as applicable:<br>**Background:** main objectives<br>**Methods:** data sources; study eligibility criteria, participants, and interventions; study appraisal; and *synthesis methods, such as network meta-analysis.*<br>**Results:** number of studies and participants identified; summary estimates with corresponding confidence/credible intervals; *treatment rankings may also be discussed. Authors may choose to summarize pairwise comparisons against a chosen treatment included in their analyses for brevity.*<br>**Discussion/Conclusions:** limitations; conclusions and implications of findings.<br>**Other:** primary source of funding; systematic review registration number with registry name.	2
**INTRODUCTION**			
Rationale	3	Describe the rationale for the review in the context of what is already known, *including mention of why a network meta-analysis has been conducted.*	3
Objectives	4	Provide an explicit statement of questions being addressed, with reference to participants, interventions, comparisons, outcomes, and study design (PICOS).	4
**METHODS**			
Protocol and registration	5	Indicate whether a review protocol exists and if and where it can be accessed (e.g., Web address); and, if available, provide registration information, including registration number.	4
Eligibility criteria	6	Specify study characteristics (e.g., PICOS, length of follow-up) and report characteristics (e.g., years considered, language, publication status) used as criteria for eligibility, giving rationale. *Clearly describe eligible treatments included in the treatment network, and note whether any have been clustered or merged into the same node (with justification).*	6
Information sources	7	Describe all information sources (e.g., databases with dates of coverage, contact with study authors to identify additional studies) in the search and date last searched.	6
Search	8	Present full electronic search strategy for at least one database, including any limits used, such that it could be repeated.	Appendix A

Study selection	9	State the process for selecting studies (i.e., screening, eligibility, included in systematic review, and, if applicable, included in the meta-analysis).	5
Data collection process	10	Describe method of data extraction from reports (e.g., piloted forms, independently, in duplicate) and any processes for obtaining and confirming data from investigators.	5
Data items	11	List and define all variables for which data were sought (e.g., PICOS, funding sources) and any assumptions and simplifications made.	5
**Geometry of the network**	**S1**	Describe methods used to explore the geometry of the treatment network under study and potential biases related to it. This should include how the evidence base has been graphically summarized for presentation, and what characteristics were compiled and used to describe the evidence base to readers.	6
Risk of bias within individual studies	12	Describe methods used for assessing risk of bias of individual studies (including specification of whether this was done at the study or outcome level), and how this information is to be used in any data synthesis.	6
Summary measures	13	State the principal summary measures (e.g., risk ratio, difference in means). *Also describe the use of additional summary measures assessed, such as treatment rankings and surface under the cumulative ranking curve (SUCRA) values, as well as modified approaches used to present summary findings from meta-analyses.*	8
Planned methods of analysis	14	Describe the methods of handling data and combining results of studies for each network meta-analysis. This should include, but not be limited to:<br>• *Handling of multi-arm trials;*<br>• *Selection of variance structure;*<br>• *Selection of prior distributions in Bayesian analyses; and*<br>• *Assessment of model fit.*	8
**Assessment of Inconsistency**	**S2**	Describe the statistical methods used to evaluate the agreement of direct and indirect evidence in the treatment network(s) studied. Describe efforts taken to address its presence when found.	7
Risk of bias across studies	15	Specify any assessment of risk of bias that may affect the cumulative evidence (e.g., publication bias, selective reporting within studies).	**7**
Additional analyses	16	Describe methods of additional analyses if done, indicating which were pre-specified. This may include, but not be limited to, the following:<br>• Sensitivity or subgroup analyses;<br>• Meta-regression analyses;<br>• *Alternative formulations of the treatment network; and*<br>• *Use of alternative prior distributions for Bayesian analyses (if applicable).*	

RESULTS†

Study selection	17	Give numbers of studies screened, assessed for eligibility, and included in the review, with reasons for exclusions at each stage, ideally with a flow diagram.	8
**Presentation of network structure**	**S3**	Provide a network graph of the included studies to enable visualization of the geometry of the treatment network.	Figure 2B, 3B, 4B, 5B
**Summary of network geometry**	**S4**	Provide a brief overview of characteristics of the treatment network. This may include commentary on the abundance of trials and randomized patients for the different interventions and pairwise comparisons in the network, gaps of evidence in the treatment network, and potential biases reflected by the network structure.	Table 1
Study characteristics	18	For each study, present characteristics for which data were extracted (e.g., study size, PICOS, follow-up period) and provide the citations.	Table 1
Risk of bias within studies	19	Present data on risk of bias of each study and, if available, any outcome level assessment.	Appendix B
Results of individual studies	20	For all outcomes considered (benefits or harms), present, for each study: 1) simple summary data for each intervention group, and 2) effect estimates and confidence intervals. *Modified approaches may be needed to deal with information from larger networks.*	Appendix B
Synthesis of results	21	Present results of each meta-analysis done, including confidence/credible intervals. *In larger networks, authors may focus on comparisons versus a particular comparator (e.g. placebo or standard care), with full findings presented in an appendix. League tables and forest plots may be considered to summarize pairwise comparisons.* If additional summary measures were explored (such as treatment rankings), these should also be presented.	Table 2D, 3D, 4D, 5D
**Exploration for inconsistency**	**S5**	Describe results from investigations of inconsistency. This may include such information as measures of model fit to compare consistency and inconsistency models, *P* values from statistical tests, or summary of inconsistency estimates from different parts of the treatment network.	Appendix B
Risk of bias across studies	22	Present results of any assessment of risk of bias across studies for the evidence base being studied.	Table 2C, 3C, 4C, 5C
Results of additional analyses	23	Give results of additional analyses, if done (e.g., sensitivity or subgroup analyses, meta-regression analyses, *alternative network geometries studied, alternative choice of prior distributions for Bayesian analyses,* and so forth).	

DISCUSSION

Summary of evidence	24	Summarize the main findings, including the strength of evidence for each main outcome; consider their relevance to key groups (e.g., healthcare providers, users, and policy-makers).	22
Limitations	25	Discuss limitations at study and outcome level (e.g., risk of bias), and at review level (e.g., incomplete retrieval of identified research, reporting bias). *Comment on the validity of*	23

		the assumptions, such as transitivity and consistency. Comment on any concerns regarding network geometry (e.g., avoidance of certain comparisons).	
Conclusions	26	Provide a general interpretation of the results in the context of other evidence, and implications for future research.	23
**FUNDING**			
Funding	27	Describe sources of funding for the systematic review and other support (e.g., supply of data); role of funders for the systematic review. This should also include information regarding whether funding has been received from manufacturers of treatments in the network and/or whether some of the authors are content experts with professional conflicts of interest that could affect use of treatments in the network.	24

PICOS = population, intervention, comparators, outcomes, study design.
* Text in italics indicateS wording specific to reporting of network meta-analyses that has been added to guidance from the PRISMA statement.
† Authors may wish to plan for use of appendices to present all relevant information in full detail for items in this section.

Comparative Efficacy of Labor Analgesia: A Network Meta-Analysis

To enable PROSPERO to focus on COVID-19 submissions, this registration record has undergone basic automated checks for eligibility and is published exactly as submitted. PROSPERO has never provided peer review, and usual checking by the PROSPERO team does not endorse content. Therefore, automatically published records should be treated as any other PROSPERO registration. Further detail is provided here.

Citation

Yiru Chen, Hongchun Chen. Comparative Efficacy of Labor Analgesia: A Network Meta-Analysis. PROSPERO 2023 CRD42023417670 Available from: https://www.crd.york.ac.uk/prospero/display_record.php?ID=CRD42023417670

Review question

What is the optimal pharmacological intervention for labor pain management? More specifically,

(1) What are the most effective drugs for maternal analgesia.

(2) Is there a difference in efficacy between single drug therapy and combination therapy for maternal analgesia.

(3) Which drugs have minimal impact on labor and have fewer side effects for maternal analgesia.

Searches

The PubMed, EMBASE, Web of Science and the Cochrane Library databases will be searched using terms such as "Labor Pain"and "epidural analgesia".

Publication period is 1970 ~ 2022.

Types of study to be included

Inclusions:

- Experimental studies that have a comparison group or condition

- Randomised

- Completed studies that are full text and published in peer-reviewed journals

Exclusions:

- Non-experimental studies

- Studies that are abstract or poster form only

- Studies that did not obtain ethics approval or IRB

- Case studies with less than 10 patients

- Studies that did not have target extraction data in the text and attachments

Condition or domain being studied

Parturients who are classified as American Society of Anesthesiologists (ASA) Class I or II.

The result can be expressed as Analgesic Efficacy.

Participants/population

Inclusion criteria:

Parturients classified as American Society of Anesthesiologists (ASA) Grade I or II, In labor and With no known fetal abnormalities.

Exclusion criteria:

Known drug allergy, Obstetric complications, Preeclampsia, Contraindications to anesthesia, Abnormal liver or kidney function, Abnormal blood function, and Drug dependence

Intervention(s), exposure(s)

A comparison of the efficacy of epidural, intrathecal, and intravenous analgesia in postpartum women, with the exclusion of drugs used for test dosing.

Comparator(s)/control

Where relevant, give details of the alternatives against which the main subject/topic of the review will be compared (e.g. another intervention or a non-exposed control group). The preferred format includes details of both inclusion and exclusion criteria.

We will include studies that have no treatment control group or condition, or that use a standard management. This may include self-control and crossover studies.

Exclusions:

- Studies that do not incorporate a comparison group or condition

Context

Main outcome(s)

Analgesic Efficacy

Measures of effect

Relative Risk

Additional outcome(s)

Duration of first stage of labor, Duration of second stage of labor, Nausea

Measures of effect

Odds Ratio or Risk Difference

Data extraction (selection and coding)

We will extract the following data from the included articles: title, author name, publication data, and number of papers, patient, study design (including grouping, blinding, etc.), drug use description (drug name and dosage), analgesic effect (VAS and other related evaluation methods), and side effects.

Risk of bias (quality) assessment

We will use the Cochrane Handbook to assess the risk of bias for all articles. The following information will be evaluated: random sequence generation, allocation concealment, blinding, incomplete outcome data, selective reporting and other bias. Assessment and evaluation will be conducted independently by two reviewers. Other two reviewers will be consulted to reconcile any disagreements. Subgroup analysis will be carried out in order to evaluate the heterogeneity of the randomized trials.

Strategy for data synthesis

All data in our study will be collected from RCT papers in which mostly descriptive synthesis will be used. If we need concrete information, we will have to ask authors for their data. The data aggregated through heterogeneity tests will be analysed by meta-analysis or sub group meta-analysis.

Analysis of subgroups or subsets

All data in our study will be collected from RCT papers in which mostly descriptive synthesis will be used. If we need specific information, we will download it from the attachment. The data aggregated through heterogeneity tests will be analysed by meta-analysis or sub group meta-analysis.

Contact details for further information

Chunhui Yuan

yuanch@zucc.edu.cn

Organisational affiliation of the review

Hangzhou City University

Review team members and their organisational affiliations

Ms Yiru Chen. Zhejiang University City College

Ms Hongchun Chen. Zhejiang University of Technology

Type and method of review

Network meta-analysis, Systematic review

Anticipated or actual start date

20 April 2023

Anticipated completion date [1 change]

20 October 2023

Funding sources/sponsors

No Funding sources/sponsors.

Conflicts of interest

Language

English

Country

China

Stage of review [1 change]

Review Completed not published

Subject index terms status

Subject indexing assigned by CRD

Subject index terms

Analgesia; Analgesics; Female; Humans; Labor, Obstetric; Pain; Pregnancy

Date of registration in PROSPERO

27 April 2023

Date of first submission

16 April 2023

Stage of review at time of this submission [1 change]

Stage	Started	Completed
Preliminary searches	Yes	Yes
Piloting of the study selection process	Yes	Yes
Formal screening of search results against eligibility criteria	Yes	Yes
Data extraction	Yes	Yes
Risk of bias (quality) assessment	Yes	Yes
Data analysis	Yes	Yes

Revision note

The article has been completed but not published.

The record owner confirms that the information they have supplied for this submission is accurate and complete and they understand that deliberate provision of inaccurate information or omission of data may be construed as scientific misconduct.

The record owner confirms that they will update the status of the review when it is completed and will add publication details in due course.

Versions

27 April 2023

27 April 2023

26 October 2023

Unique ID	Reviewer	Study ID	Reference	Experimental	Comparator
Tveit 2012	cyr	Tveit 2012			
Evron 2005		Evron 2005			
Douma 2010		Douma 2010			
Stocki 2014		Stocki 2014			
Douma 2011		Douma 2011			
Ismail 2012		Ismail 2012			
Jia 2020		Jia 2020			
Rezk 2015		Rezk 2015			
Nunes 2019		Nunes 2019			
Zhao 2017		Zhao 2017			
Wang 2018		Wang 2018			
Karadjova 2019		Karadjova 2019			
Li 2020		Li 2020			
Cheng 2019		Cheng 2019			
Wu 2022		Wu 2022			

Outcome	Result	Aim	Effect of adhering to intervention?	Weight
		assignment to intervention	NA	1
		assignment to intervention	NA	1
		assignment to intervention	NA	1
		assignment to intervention	NA	1
		assignment to intervention	NA	1
		assignment to intervention	NA	1
		assignment to intervention	NA	1
		assignment to intervention	NA	1
		assignment to intervention	NA	1
		assignment to intervention	NA	1
		assignment to intervention	NA	1
		assignment to intervention	NA	1
		assignment to intervention	NA	1
		assignment to intervention	NA	1
		assignment to intervention	NA	1

Randomization process	Comment for randomization process	Deviations from intended interventions	Comment for deviations from intended interventions	Missing outcome data
Low		Some concerns		Low
Low		Low		Low
Low		Low		Low
Some concerns		Low		Low
Low		Low		Some concerns
Low		Low		Low
Some concerns		Some concerns		Low
Low		Low		Low
Low		Low		Low
Low		Low		Low
Some concerns		Some concerns		Low
Low		Some concerns		Low
Low		Low		Low
Low		Low		Low
Some concerns		Some concerns		Low

Comment for missing outcome data	Measurement of the outcome	Comment for measurement of the outcome	Selection of the reported result	Comment for selection of the reported result
	Low		Low	
	Low		Low	
	Low		Low	
	Low		Low	
	Some concerns		Low	
	Low		Low	
	Some concerns		Low	
	Low		Low	
	Low		Low	
	Low		Low	
	Some concerns		Low	
	Some concerns		Low	
	Low		Low	
	Low		Low	
	Some concerns		Low	

Overall Bias	Comment for overall bias
Some concerns	
Low	
Low	
Some concerns	
Some concerns	
Low	
Some concerns	
Low	
Low	
Low	
Some concerns	
Some concerns	
Low	
Low	
Some concerns	

After pressing this button, results
summary table (left table) will ove
the results in 'Results' tab. This he

in the
erwrite
lps you to

	Randomization process	Deviations from intended interventions	Mising outcome data
Assignment to intervention (the 'intention-to-treat' effect)			
Total number of study = 15			
Low risk	73.3	66.7	93.3
Some concerns	26.7	33.3	6.7
High risk	0	0	0
Adhering to intervention (the 'per-protocol' effect)			
Total number of study = 0			
Low risk			
Some concerns			
High risk			

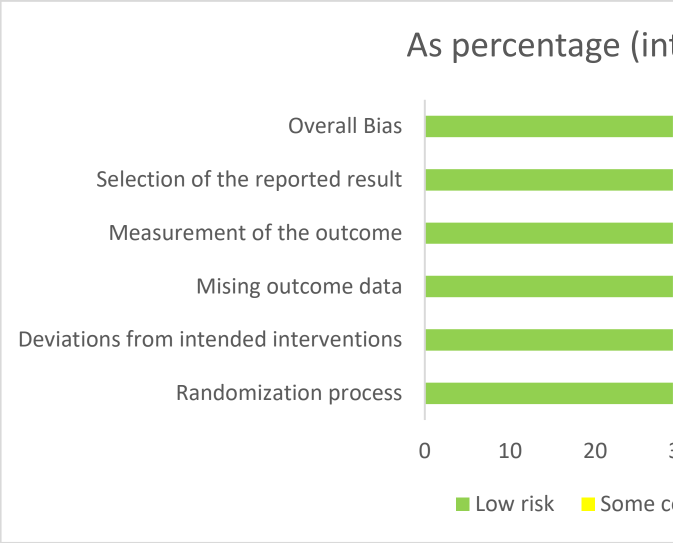

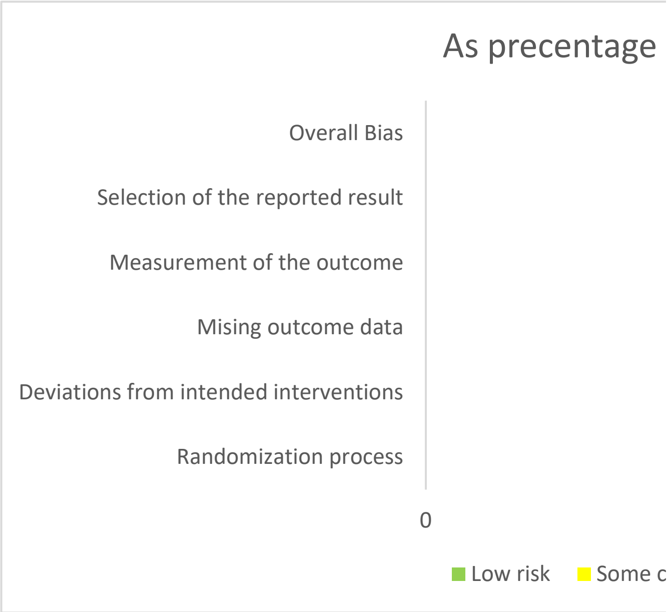

Measurement of the outcome	Selection of the reported result	Overall Bias
66.7	100	53.3
33.3	0	46.7
0	0	0

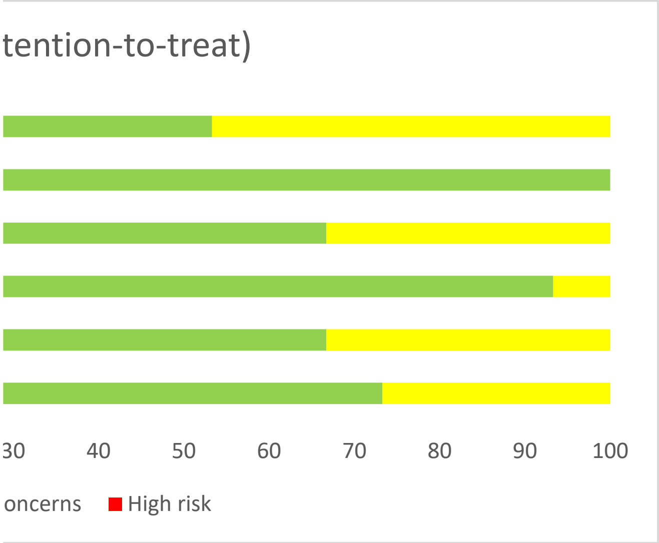

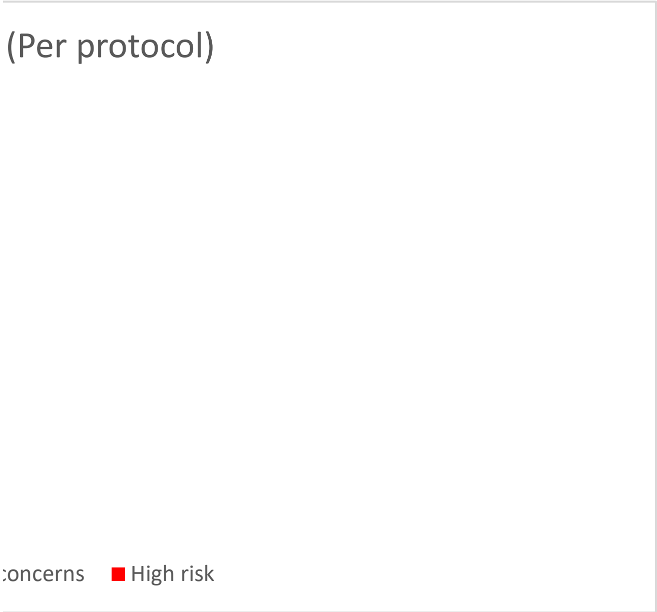

Supplement: S3 File — (PDF) [file pone.0303174.s003.pdf]
